# Of mites and cyanide: Rapid spider mite adaptation to Arabidopsis defense metabolites

**DOI:** 10.1093/plphys/kiac247

**Published:** 2022-05-27

**Authors:** Rachel E Kerwin

**Affiliations:** Department of Biochemistry and Molecular Biology, Michigan State University, East Lansing, Michigan 48824, USA

To get a meal, herbivores must contend with the extensive chemical arsenal of plants, an arsenal that collectively exceeds 200,000 compounds (Pichersky and Gang, 2000). While some herbivores opt to specialize on one or a few hosts, the two-spotted spider mite (*Tetranychus urticae*) is an opportunistic feeder able to utilize an astounding array of plant species (Rioja et al., 2017). Many generalist herbivores enjoy a broad host range because they can quickly acclimate to a diverse portfolio of plant defenses. In contrast, individual spider mite populations are locally adapted to just one or a few species but can evolve counter-measures to combat the defense compounds of a different plant within a few generations (Salehipourshirazi et al., 2021). This rapid specialization enables spider mites to serially colonize additional plant hosts. However, the nature of adapted counter defense traits and the mechanisms through which they evolve have remained elusive.

Although spider mites are able to colonize over 1,000 plant species, Arabidopsis (*Arabidopsis thaliana*) is not a typical host. The defense repertoire of Arabidopsis includes amino acid-derived glucosinolates. Once synthesized, glucosinolates, in their glycoside form, are stored in plant cells as innocuous precursors. Upon tissue disruption caused by herbivore attack, for example, these glycosidic precursors are hydrolyzed, releasing bioactive compounds, including isothiocyanates or nitriles (Halkier and Gershenzon, 2006). In its chemical defense against spider mites, Arabidopsis primarily uses indole glucosinolates, derived from the amino acid tryptophan (Zhurov et al., 2014; Figure 1A).

**Figure 1:**
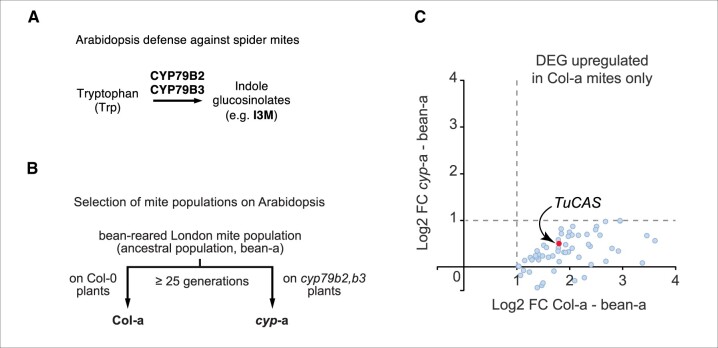
Inducibility of *TuCAS*, encoding β-cyanoalanine synthase (CAS) from two-spotted spider mite (*T. urticae*), enables cyanide tolerance and underlies adaptation to Arabidopsis (*A. thaliana*). A, Tryptophan-derived indole glucosinolates are Arabidopsis defense metabolites that effectively deter spider mite herbivory. B, An ancestral spider mite population adapted to the common bean, *P. vulgaris* (i.e. ancestral population, bean-adapted [bean-a]), was transferred to two Arabidopsis genotypes: either the well-defended wild-type Col-0 or the vulnerable cytochrome P450 CYP79B2 CYP79B3 double mutant *cyp97b2 cyp79b3* (*cyp79b2,b3*) that lacks the ability to synthesize indole glucosinolates. After ∼25 generations (∼18 months), 2 spider mite genotypes evolved that were well-suited to their new plant hosts: Col-0-adapted (Col-a) and *cyp79b2 cyp79b3*-adapted (*cyp*-a). C, Fifty-nine transcripts are significantly upregulated (log2 fold-change >1, *P*-value < 0.05) in Col-0-adapted versus bean-adapted mites, but not in *cyp79b2 cyp79b3*-adapted versus bean-adapted mites. The *TuCAS* transcript is indicated by arrow. Transcriptomics experiment was performed in three biological replicates (*n* = 3). DEG, differentially expressed genes. I3M, indole-3-ylmethyl glucosinolate. Modified from Dixit et al. (2022), Figure 1.

Spider mites, however, are no slouches in this chemical arms race and can adapt readily to different plant defense compounds. For example, Salehipourshirazi et al. (2021) transferred a spider mite population adapted to the common bean (*Phaseolus vulgaris*) to two Arabidopsis genotypes: either the well-defended wild-type Col-0 (Col-0) or the vulnerable cytochrome P450 CYP79B2 CYP79B3 double mutant (*cyp79b2 cyp79b3*) that lacks the ability to synthesize indole glucosinolates (Figure 1B). After suffering initially, the spider mites adapted to their newly colonized hosts in ∼25 generations (∼18 months), yielding Col-0-adapted and *cyp79b2 cyp79b3*-adapted populations that are genetically distinct from their bean-adapted ancestors (Figure 1B).

In this issue of *Plant Physiology*, Dixit et al. (2022) employed an elegant transcriptomics experiment to identify genetic and functional changes underlying spider mite adaptation to Arabidopsis. Bean-adapted, Col-0-adapted, and *cyp79b2 cyp79b3*-adapted mites were reared on bean, Col-0, and *cyp79b2 cyp79b3* plants, after which transcriptomes of the nine mite sample groups (three mite genotypes × three plant genotypes) were sequenced in triplicate, enabling the authors to quantify expression level and coding sequence differences. The authors identified 59 transcripts more highly expressed in Col-0-adapted mites than *cyp79b2 cyp79b3*-adapted and bean-adapted mites, including several involved in detoxification of foreign metabolites (i.e. xenobiotics) (Figure 1C).

One of the transcripts enriched in Col-0-adapted mites, *TuCAS*, which encodes β-cyanoalanine synthase (CAS), stood out to the authors. Baseline *TuCAS* expression was uniform across the three mite genotypes when fed bean leaves. However, when mites were reared on Arabidopsis leaves (either Col-0 or *cyp79b2 cyp79b3*), *TuCAS* expression was induced in Col-0-adapted mites, but not bean-adapted or *cyp79b2 cyp79b3*-adapted mites. The authors puzzled over why TuCAS would be induced in Arabidopsis-fed spider mites. Due to its ability to detoxify cyanide, TuCAS is required for spider mites to handle consumption of cyanide-releasing cyanogenic glycosides, but these defense compounds are not found in Arabidopsis. What benefit could higher *TuCAS* expression possibly confer to spider mites feeding on Arabidopsis leaves? Is *TuCAS* inducibility actually involved in spider mite adaptation to Arabidopsis or is it simply a byproduct of evolution? To answer these questions, the authors needed to understand the role *TuCAS* plays, if any, in enabling spider mites to feed on Arabidopsis.

To investigate the function of *TuCAS*, the authors employed RNA interference (RNAi) to silence its expression in Col-0-adapted mites. *TuCAS*-silenced Col-0-adapted mites suffered decreased fecundity compared with untreated Col-0-adapted mites when reared on Col-0 leaves but suffered no ill effects when reared on bean leaves. These results demonstrated that *TuCAS* enables Col-0-adapted mites to successfully feed on Arabidopsis, though the “why” remained elusive. Does the inducible Col-0-adapted *TuCAS* allele (i.e. gene variant) confers a greater cyanide detoxification capacity? To address this question, they challenged mites with increasing concentrations of potassium cyanide and found that Col-0-adapted mites were, in fact, more cyanide-tolerant than their bean-adapted ancestors.

Does that mean Arabidopsis releases cyanide upon spider mite herbivory? If so, what is the source? To test these questions, the authors measured leaf cyanide levels directly. They found that cyanide levels were approximately four times greater in untreated leaves from Col-0 than indole glucosinolate-deficient *cyp79b2 cyp79b3*, indicating that indole glucosinolates are a source of cyanide in Arabidopsis leaves.

Further, upon spider mite feeding, leaf cyanide levels increased 50% in Col-0, but did not change in *cyp79b2 cyp79b3*. Leaves were assayed after 2 h of spider mite feeding, when hydrolysis (i.e. activation) of indole glucosinolates has been triggered but herbivory-induced biosynthesis has not yet occurred (Widemann et al., 2021). To further investigate the relationship between indole glucosinolate activation and cyanide production, the authors treated *cyp79b2 cyp79b3* leaves with indole glucosinolates for 24 h then performed spider mite herbivory assays. They found that exogenous indole glucosinolate supplementation greatly boosted leaf cyanide levels, with or without subsequent mite herbivory.

Together, these results reveal three things: (i) indole glucosinolates provide a source of cyanide in the leaves of well-defended Arabidopsis Col-0, (ii) cyanide may be released as a byproduct of indole glucosinolate activation, and (iii) spider mite feeding may favor indole glucosinolate activation-dependent cyanide release. Future experiments are needed to determine the exact conditions under which indole glucosinolate-dependent cyanide release occurs.

This study investigated the tempo and mode of evolution by exploring the adaptive changes that occur as spider mites colonize a plant host. While adaptation typically occurs over millions of years, it can also happen in just a few generations, as exemplified by these serial specialists. Dixit and colleagues demonstrated that increased cyanide tolerance, conferred by an inducible *TuCAS* allele, rapidly arose in spider mites during adaptation to well-defended Arabidopsis Col-0. These findings support the hypothesis that changes in gene expression regulation underlie rapid, repeated bouts of adaptation (López-Maury et al., 2008).

*Conflict of interest statement*. None declared.

## References

[kiac247-B1] Dixit S , Widemann E, Bensoussan N, Salehipourshirazi G, Bruinsma K, Milojevic M, Shukla A, Romero LC, Zhurov V, Bernards MA, et al (2022) β-cyanoalanine synthase protects mites against Arabidopsis defenses. Plant Physiol **189**: 1956–1970 10.1093/plphys/kiac147 PMC9342966 35348790

[kiac247-B2] Halkier BA , Gershenzon J (2006) Biology and biochemistry of glucosinolates. Annu Rev Plant Biol 57: 303–333 16669764 10.1146/annurev.arplant.57.032905.105228

[kiac247-B3] López-Maury L , Marguerat S, Bähler J (2008) Tuning gene expression to changing environments: From rapid responses to evolutionary adaptation. Nat Rev Genet 9: 583–593 18591982 10.1038/nrg2398

[kiac247-B4] Pichersky E , Gang DR (2000) Genetics and biochemistry of secondary metabolites in plants: An evolutionary perspective. Trends Plant Sci 5: 439–445. 11044721 10.1016/s1360-1385(00)01741-6

[kiac247-B5] Rioja C , Zhurov V, Bruinsma K, Grbic M, Grbic V (2017). Plant–herbivore interactions: A case of an extreme generalist, the two-spotted spider mite *Tetranychus urticae*. Mol Plant Microbe Interact 30: 935–945 28857675 10.1094/MPMI-07-17-0168-CR

[kiac247-B6] Salehipourshirazi G, Bruinsma K, Ratlamwala H, Dixit S, Arbona V, Widemann E, Milojevic M, Jin P, Bensoussan N, Gómez-Cadenas A et al. (2021) Rapid specialization of counter defenses enables two-spotted spider mite to adapt to novel plant hosts. Plant Physiol 187: 2608–2622 34618096 10.1093/plphys/kiab412 PMC8644343

[kiac247-B7] Widemann E , Bruinsma K, Walshe-Roussel B, Rioja C, Arbona V, Saha RK, Letwin D, Zhurov V, Gómez-Cadenas A, Bernards MA, et al (2021) Multiple indole glucosinolates and myrosinases defend Arabidopsis against *Tetranychus urticae* herbivory. Plant Physiol 187: 116–132 34618148 10.1093/plphys/kiab247 PMC8418412

[kiac247-B8] Zhurov Vl, Navarro M, Bruinsma KA, , Arbona V, Santamaria ME, Cazaux M, Wybouw N, Osborne EJ, , Ens C, Rioja C et al. (2014) Reciprocal responses in the interaction between Arabidopsis and the cell-content-feeding chelicerate herbivore spider mite. Plant Physiol 164: 384–399 24285850 10.1104/pp.113.231555 PMC3875816

